# Gefitinib and fostamatinib target EGFR and SYK to attenuate silicosis: a multi-omics study with drug exploration

**DOI:** 10.1038/s41392-022-00959-3

**Published:** 2022-05-13

**Authors:** Mingyao Wang, Zhe Zhang, Jiangfeng Liu, Meiyue Song, Tiantian Zhang, Yiling Chen, Huiyuan Hu, Peiran Yang, Bolun Li, Xiaomin Song, Junling Pang, Yanjiang Xing, Zhujie Cao, Wenjun Guo, Hao Yang, Jing Wang, Juntao Yang, Chen Wang

**Affiliations:** 1 State Key Laboratory of Medical Molecular Biology, Institute of Basic Medical Sciences Chinese Academy of Medical Sciences, School of Basic Medicine Peking Union Medical College, Beijing, 100005 China; 2 Laboratory of Stem Cell and Tissue Engineering, Orthopedic Research Institute, State Key Laboratory of Biotherapy and Cancer Center, West China Hospital, Sichuan University and Collaborative Innovation Center of Biotherapy, Chengdu, 610041 China; 3 Department of Pulmonary and Critical Care Medicine, The First Hospital of Shanxi Medical University, Taiyuan, 030001 China; 4 NHC Key Laboratory of Pneumoconiosis, Taiyuan, 030001 China; 5 Shanxi Province Key Laboratory of Respiratory Disease, Taiyuan, 030001 China; 6 Department of Pulmonary and Critical Care Medicine, Center of Respiratory Medicine, China-Japan Friendship Hospital, Beijing, 100029 China; 7 Department of Respiratory and Critical Care Medicine, The First Affiliated Hospital of Xi’an Jiao tong University, Xi’an, 710061 China; 8 Key Lab of Transplant Engineering and Immunology, MOH; Regenerative Medical Research Center, West China Hospital, Sichuan University, Chengdu, 610041 China

**Keywords:** Translational research, Respiratory tract diseases, Molecular medicine

## Abstract

Silicosis is the most prevalent and fatal occupational disease with no effective therapeutics, and currently used drugs cannot reverse the disease progress. Worse still, there are still challenges to be addressed to fully decipher the intricated pathogenesis. Thus, specifying the essential mechanisms and targets in silicosis progression then exploring anti-silicosis pharmacuticals are desperately needed. In this work, multi-omics atlas was constructed to depict the pivotal abnormalities of silicosis and develop targeted agents. By utilizing an unbiased and time-resolved analysis of the transcriptome, proteome and phosphoproteome of a silicosis mouse model, we have verified the significant differences in transcript, protein, kinase activity and signaling pathway level during silicosis progression, in which the importance of essential biological processes such as macrophage activation, chemotaxis, immune cell recruitment and chronic inflammation were emphasized. Notably, the phosphorylation of EGFR (p-EGFR) and SYK (p-SYK) were identified as potential therapeutic targets in the progression of silicosis. To inhibit and validate these targets, we tested fostamatinib (targeting SYK) and Gefitinib (targeting EGFR), and both drugs effectively ameliorated pulmonary dysfunction and inhibited the progression of inflammation and fibrosis. Overall, our drug discovery with multi-omics approach provides novel and viable therapeutic strategies for the treatment of silicosis.

## Introduction

Silicosis is a progressive and irreversible disease, in which the retained crystalline silica results in continuous inflammation, tissue damage, and fibrosis, eventually leading to respiratory failure and death.^1^ Exacerbating this issue is that no curative drugs are available for silicosis.^2–4^ However, pharmaceutical development for silicosis remains challenging due to unclear molecular mechanism.^1,4,5^ Therefore, anti-silicosis drug discovery counts on comprehensively deciphering and delving the aberrant pathological landscape of silicosis.

As yet, most mechanistic studies of silicosis have been restricted to a few genes and specific pathways, leaving the etiopathology of silicosis poorly defined. Current studies suggest that the pathogenesis of silicosis involves persistent immune responses and cytokine secretion induced by dust inhalation, which can be divided into the following five pathways^6–9^: direct damage caused by dust particles in alveolar tissue; oxidative stress caused by reactive oxygen species (ROS) and reactive nitrogen species (RNS); inflammation activated by NF-κB, AP-1, and other inflammatory factors; fibrosis promoted by transforming growth factor beta (TGF-β), tumor necrosis factor alpha (TNF-α), and matrix metalloproteinases (MMPS); caspase-mediated apoptosis induced by dust particles and related inflammatory factors. Despite the known contribution of these pathways, the molecular mechanisms and key regulators underlying these processes still remained unclear,^10,11^ hampering drugs discovery for the treatment of silicosis.

In the era of systems medicine, multi-omics technologies are well-known to accelerate the success of drug discovery.^12–14^ Recently, multi-omics have become pillars of better understanding mechanisms and fostering therapeutics of various lung diseases, such as chronic obstructive pulmonary disease (COPD),^15–17^ idiopathic pulmonary fibrosis (IPF)^18^ and silicosis.^19,20^ Specifically, our recent study combining transcriptomic and metabolomic datasets revealed that ramatroban attenuated silicosis progression by suppressing the arachidonic acid pathway.^19^ In addition, substantial evidence has proven that phosphorylation of tyrosine kinases (TKIs) played a vital role in the pathogenesis of IPF, and nintedanib, a TKI inhibitor, brought promising outcomes for IPF.^3,21^ Taken together, the proteome and transcriptome can reveal differentially expressed features and essential pathways in silicosis progression, while the phosphoproteome provides major post-translational modification changes that regulate the expression of downstream transcripts and proteins. With combined analysis of the transcriptome, proteome and phosphoproteome, potential kinase-targeted therapeutic candidates in key signaling pathways can be effectively identified and applied for silicosis treatments.

In this study, we first constructed a silicosis mouse model of different stages to perform transcriptomic, proteomic, and phosphoproteomic analysis. Next, multi-omics datasets were integrated to analyze dynamic progression of silicosis with lung tissues of silica-injured mice. Time-resolved and comprehensive three-omic landscape was generated and revealed the aberrant pathways at mRNA, protein, and protein phosphorylation levels. We further identified key signaling pathways and promising drug targets in silicosis, which may be exploited in future clinical practices.

## Results

### Comprehensive characterization of the transcriptome, proteome, and phosphoproteome of silicosis mouse lungs

In order to assess the altered transcripts and proteins in silicosis, we utilized a silica-exposed mouse model^22^ and collected lung tissues from mice at different time points (2W, 4W, 6W, 10W) after silica exposure (Supplementary Fig. 1a). To assess the severity of silicosis, we measured the degree of inflammation (Supplementary Fig. 1b) and fibrosis (Supplementary Fig. 1c) in these mice. As shown in Supplementary Fig. 1, pulmonary inflammation and fibrosis consistently worsened during silicosis progression (Supplementary Fig. 1b, c).

**Fig. 1:**
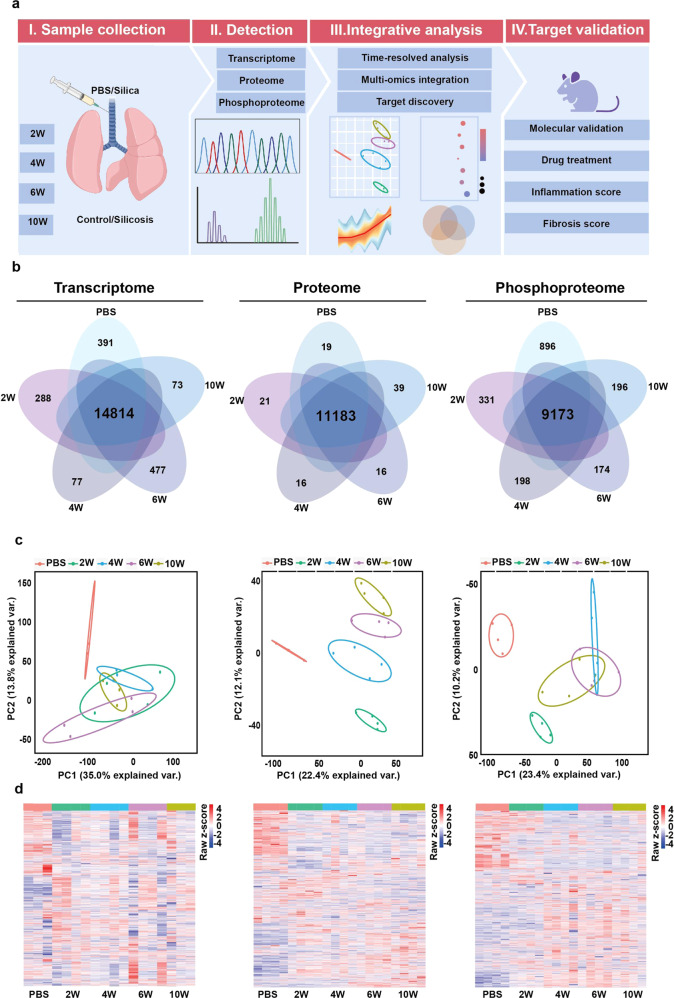
Multi-omics profiling of the silicosis mouse lungs. **a** The flow chart depicted the experimental procedures and data analysis workflow. **b** Venn diagrams showed the overlapped features including 4,745 mRNA, 11,294 proteins and 10,968 phosphosites among samples collected from silicosis model in different stages. **c** Principle component analysis (PCA) scatter plots were performed with all identified features to explore the largest sources of variance within each omics dataset. **d** Heatmaps of differentially expressed features in transcriptome, proteome, and phosphoproteome. 2W, 2 weeks; 4W, 4 weeks; 6W, 6 weeks, 10W, 10 weeks. The color bars in the heatmaps right represented the gene expression level (blue, downregulation and red, upregulation)

Using the lung tissues from silicosis mice, we performed integrative analysis of transcriptomic, proteomic, and phosphoproteomic data as described in the Methods (Fig. 1a). Overall, we identified 22,046 transcripts, 11,836 proteins (Supplementary Fig. 2) and 16,399 phosphosites (Supplementary Fig. 3), where 14,814 transcripts, 11,183 proteins and 9,173 phosphosites were shared among the five groups (Silica_2W, Silica_4W, Silica_6W, Silica_10W and PBS) (Fig. 1b). Principle component analysis revealed that transcripts, proteins, and phosphosites (Fig. 1c) were located in different clusters corresponding to the experimental groups, clearly separating the PBS-treated controls and silica-exposed models. The similarity and heterogeneity of samples from different groups in terms of transcriptome, proteome and phosphoproteome were shown in the heatmaps (Fig. 1d).

### Profiling dynamic landscapes of multi-omics changes in silicosis progression

**Fig. 2:**
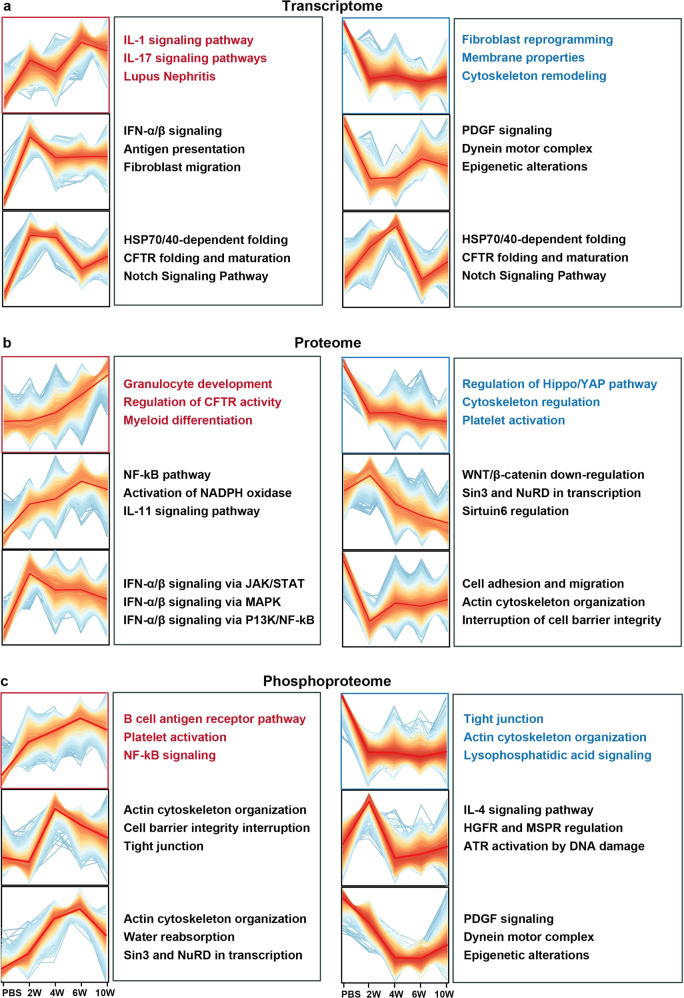
Trend analysis of silicosis progression in five groups (PBS, 2W, 4W, 6W, 10W). **a** 4340 mRNA, **b** 4482 proteins, and **c** 5052 phospho sites were clustered with fuzzy c-means clustering into 6 discrete expression clusters respectively to illustrate the relative expression changes of the transcriptomic, proteomic and phosphoproteomic data along the progression of the disease in mouse model, and the top 3 pathways enriched for each cluster in metacore were provided alongside the clusters. Key pathways highlighted in red were proposed to be consistently upregulated in silicosis among multi-omics datasets, while essential pathways highlighted in blue proposed to be consistently downregulated in silicosis among multi-omics datasets. 2W, 2 weeks; 4W, 4 weeks; 6W, 6 weeks, 10W, 10 weeks

The bioinformatic analysis was performed on the dynamic changes of features (including transcripts in transcriptome, proteins in proteome, and phosphosites in phosphoproteome) in silicosis progression using the R package Mfuzz. Various dynamic patterns were observed in the omics data at different stages as shown in Fig. 2, indicating complex changes involving a large number of pathways in silicosis progression. Different clusters were used for pathway enrichment with metacore. Transcriptomic analysis revealed that the interleukin-related responses (such as IL-1, IL-11, and IL-17 signaling pathways) were continuously upregulated, while fibroblast reprogramming and extracellular membrane remodeling were consistently downregulated (Fig. 2a). The enrichment results of proteomic data also showed the continuous upregulation of inflammation responses (such as granulocyte development and differentiation as well as CFTR activity), and suggested the downregulation of cytoskeleton regulation and platelet activation (Fig. 2b). At the phosphorylation level, enrichment results suggested that NF-kB-mediated inflammation was persistently activated, while cytoskeleton remodeling as well as lysophosphatidic acid signaling were depressed (Fig. 2c). Though the differentially expressed features in three-omic datasets participated in diverse pathways, these features were found in some key signaling pathways such as inflammatory response and interstitial fibrosis regulation.

**Fig. 3:**
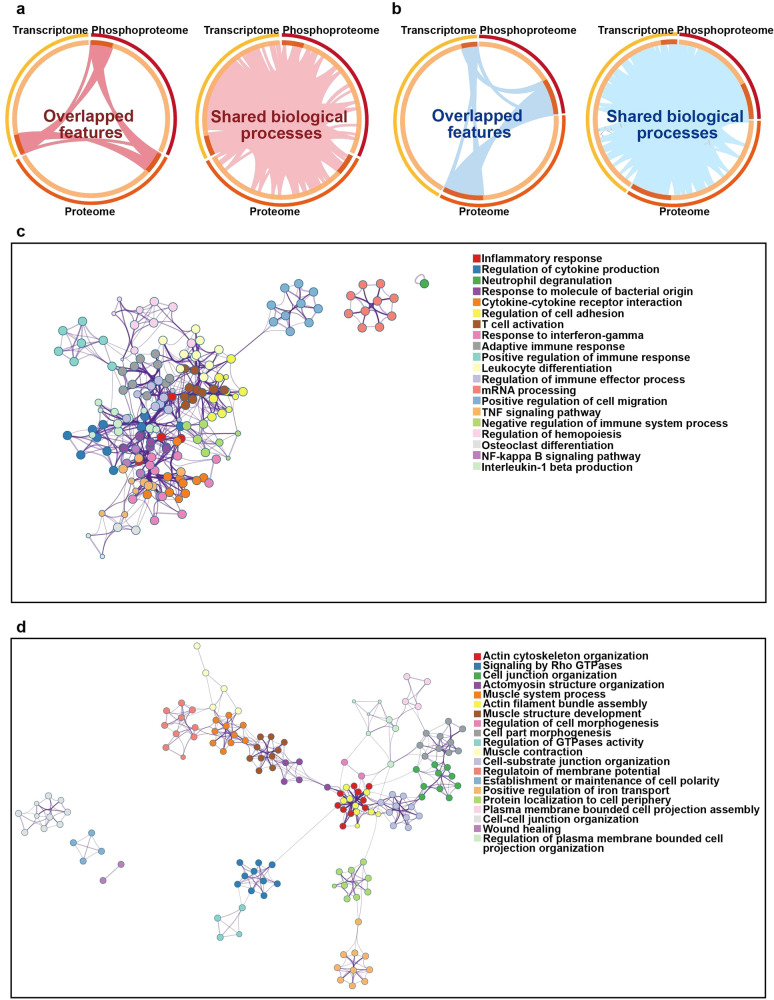
Visualization of meta-analysis results based on transcriptome, proteome, and phosphoproteome features. **a** Circos plot showed the overlap features and the overlap biological processes based on Gene Ontology between three lists (up-regulated feature listed in three omics). Dark red lines linked the same features that were shared by multiple feature lists. Light red lines linked the features which were annotated in same functional terms. **b** Circos plot showed the overlap genes and functional annotations between three lists (downregulated feature lists in three omics). Dark blue lines linked the same features that were shared by multiple feature lists. Light blue lines linked the features which were annotated in same functional terms. Enrichment network visualization for results from **c** three upregulated feature lists and **d** three downregulated feature lists, where nodes were represented by circles colored by their biological processes. The ellipses in different colors represented the proteins in different signaling pathways

To further leverage multi-omics datasets, we utilized combined analysis below for promising therapeutic target identification. Circos^23^ was first applied to reveal the correlation of features or their annotation among different omics datasets. As shown in Fig. 3a and b, only few features overlapped directly among three omics datasets while their functional annotation showed extensive overlap. To pinpoint the common pathways involving consistently up or downregulated features, interaction enrichment analysis was applied. Connected network components of upregulated features were identified in Fig. 3c, including inflammatory response, regulation of cytokines production and neutrophil degranulation, *etc*. For downregulated features, the involved network included actin cytoskeleton organization, Rho GTPase signaling, and cell junction organization (Fig. 3d). Collectively, we noticed the persistently changed pathways in high consistence with analysis data above, such as inflammatory responses and tissue remodeling in silicosis progression, which was essential for the identification of key mediators and drug targets.

### Analysis of upregulated pathways revealed EGFR and SYK as potential drug targets for silicosis

**Fig. 4:**
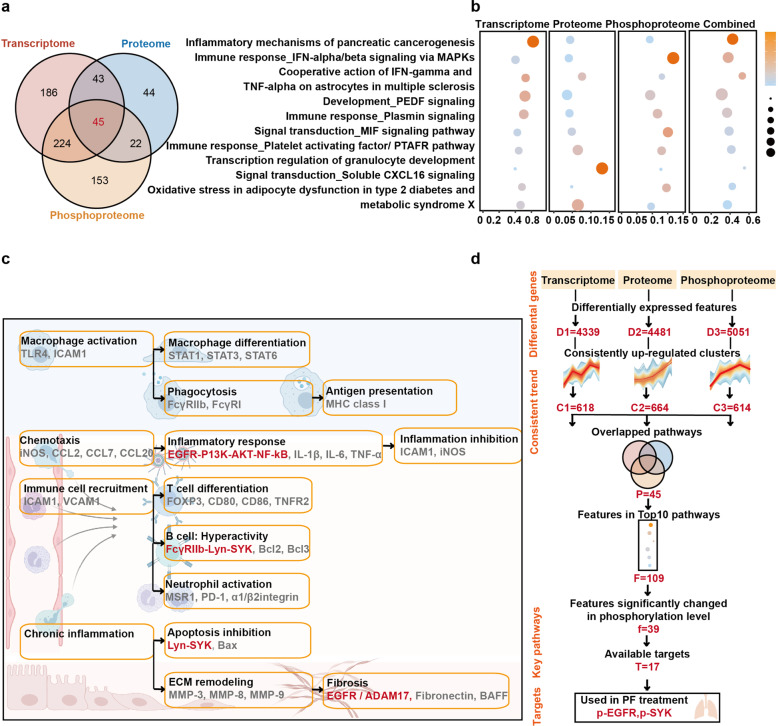
Identification of drug target in upregulated pathways with metacore. **a** Shared pathways of the upregulated modules above were obtained from metacore enrichment, with the cutoff adjusted *p* value < 0.05. **b** Bubble plot of the top 10 shared pathways in (**a**), the color represented adjusted *p-value* and bubble size represented counts of genes in the top 10 enriched pathways. **c** Illustration of the proposed underlying mechanisms involved in silicosis progression based on evidence from the multi-omics enrichment analysis. Key pathways and features highlighted in red were proposed to be important in silicosis. **d** Diagram of multi-omics data analysis and therapeutic target screening. D, differentially expressed features; C, consistently upregulated clusters; P, overlapped pathways; F, features in top 10 pathways; f, features significantly changed in phosphorylation; T, available targets

To explore novel targets for silicosis drug discovery, features with continuously changing patterns based on previous analysis results (Fig. 2) were used for further analysis with metacore. As shown in Supplementary Fig. S4, 16 overlapped pathways were enriched among downregulated features from multi-omic dataset, in which cardiac hypertrophy and macroautophagy were the main downregulated processes. Besides, the upregulated features among three omics datasets were also studied using the same method. The results showed that 498 pathways were significantly enriched in transcriptome, 154 pathways were significantly enriched in proteome and 444 pathways were significantly enriched in phosphoproteome. We found 45 pathways in common (Fig. 4a), and these pathways were mainly associated with immune responses (such as IFN signaling pathway via MAPKs and plasmin signaling) (Fig. 4b). Given that it is generally easier to suppress upregulated features with inhibitory drugs than to over-express or activate downregulated targets, we chose the upregulated features for further combined analysis and drug target identification in the drug profiling system.

We next reordered our enriched pathways with weighted *p-value* (products of *p* values enriched in three omics datasets). In order to identify novel and important drug targets, we summarized and evaluated the key regulators on our pathway maps (Fig. 4c). The functional convergence among essential multi-omics signatures in silicosis progression including macrophage activation, chemotaxis, immune cell recruitment and chronic inflammation, which were consistent with our above analysis results and suggested key pathways and available targets for further exploration. Among these pathways, the EGFR/ADAM17 axis^24^ and EGFR/AKT activity^25^ were reported to be key pathways in lung fibrosis, whereas the FcγIIb-Lyn-Syk axis was known to play significant roles in regulating macrophage activities.^26^

Among the reordered results, 85 features were included in top 10 pathways, in which existed 39 features significantly altered in phosphorylation level. Additionally, the downregulated features among three omics datasets were also studied using the same method (Supplementary Fig. 4). Sixteen overlapped pathways were identified, and cardiac hypertrophy and macroautophagy were the main downregulated processes. It was easier to suppress upregulated features with drugs than to over-express or activate downregulated ones, so we chose the upregulated features for further drug targets identification in drug profiling system. With drug target screening in DrugBank,^27^ 17 available phospho-targets with FDA-approved drugs were identified, of which p-EGFR and p-SYK inhibitors have been previously applied in pulmonary fibrosis treatment.^20–22^ These results indicated that p-EGFR and p-SYK might be potential targets for silicosis treatment (Fig. 4d).

In our results, p-EGFR and p-SYK consistently increased with silicosis progression in the phosphoproteome data. The mRNA and protein levels of these two targets also showed unstable upregulation (Supplementary Fig. 5). These results indicated that p-EGFR and p-SYK might be potential targets for silicosis treatment (Fig. 4d).

**Fig. 5:**
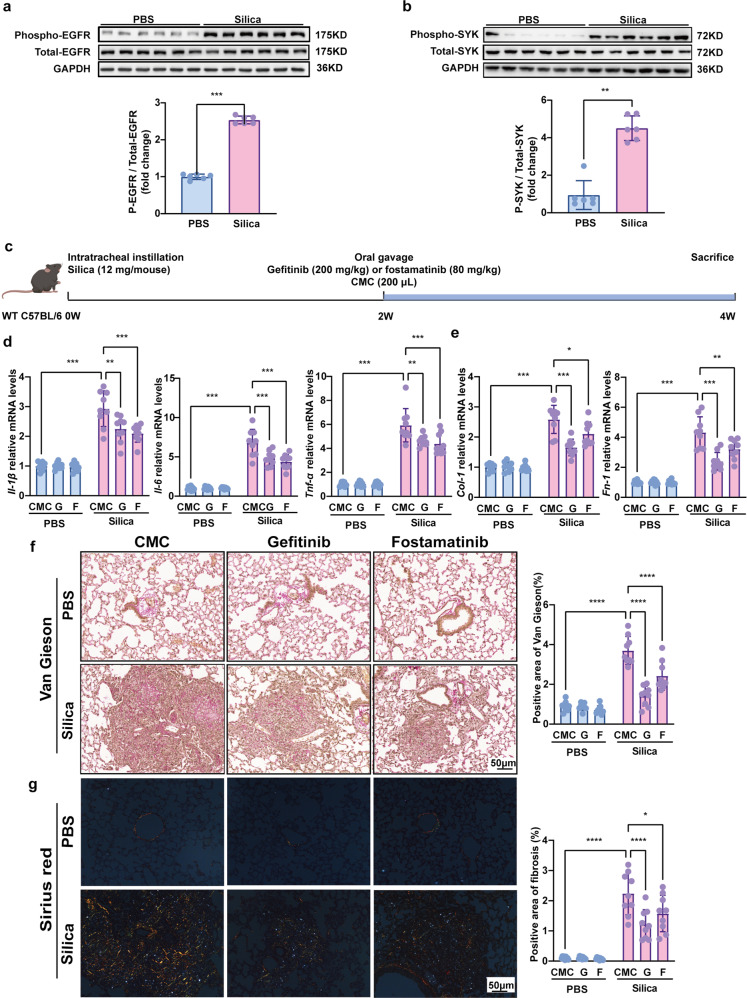
Gefitinib and fostamatinib treatment suppressed pulmonary inflammation and fibrosis in silicosis mice. Western blot analysis of **a** EGFR and **b** SYK expression and phosphorylation with silicosis and control mouse lung tissues. **c** Schematic diagram of gefitinib and fostamatinib treatment in silicosis mice. The relative mRNA levels of **d** *Il-1β*, *Il-6*, *Tnf-α*, **e** *Col-1* and *Fn-1* in lung tissues. Representative images of **f** Van Gieson, and the scale bar indicates 50 μm. Quantification of pulmonary inflammation and fibrosis in (**f**). All quantitative results were presented as mean ± SD. All the experimental groups in panel d-g were compared by a two-way ANOVA followed by Bonferroni’ s multiple comparisons test, ns: no significance, PBS group: *n* = 9 each group; Silica-exposed group: *n* = 9 each group. *p < 0.05, **p < 0.01, ***p < 0.001, ****p < 0.0001. PBS phosphate-buffered saline, G gefitinib, F fostamatinib. 2W, 2 weeks; 4W, 4 weeks

In order to further validate the results of multi-omics analysis, we first checked the expression of EGFR and SYK at protein and phosphorylation levels. Compared to control mice, lung tissues from mice exposed to 12 mg/kg silica for 6 weeks exhibited elevated phosphorylation level of EGFR and SYK, while the total protein expression of EGFR and SYK remained unchanged (Fig. 5a, b). These data implied that p-EGFR and p-SYK might play pivotal roles in stages of silicosis.

### Gefitinib and fostamatinib suppressed silicosis progression in mice

Gefitinib is an FDA-approved selective inhibitor of EGFR TKI,^28^ which is initially used as an effective treatment for advanced non-small cell lung cancer (NSCLC).^29,30^ Gefitinib was applied at a dose of 250 mg daily in lung adenocarcinoma patients,^31^ which was equivalent to 32.5 mg/kg in mice. Additionally, several preclinical studies reported that lung fibrosis progression was depressed in bleomycin and radiation resulted PF-ILD^21,32,33^ after gefitinib (200 mg/kg) treatment. Similarly, fostamatinib is also a small molecule TKI (targeting SYK), which was approved by FDA for refractory immune thrombocytopenia treatment at first.^34^ Clinically, fostamatinib was applied as doses of 150 mg two times each day (conversed as 39 mg/kg in mice) in severe COVID-19 patients with pulmonary fibrosis.^35^ Meanwhile, recent studies seemed to reach a consensus that 80 mg/kg fostamatinib showed highly antifibrotic potent in various fibrosis mouse models.^3,36–38^ Therefore, in accordance with previous studies, we selected gefitinib (200 mg/kg)^39^ and fostamatinib (80 mg/kg)^40^ as promising candidate drugs to respectively inhibit p-EGFR and p-SYK in the following silicosis treatment experiments (Fig. 5c). Related dose researches of gefitinib and fostamatinib must be well considered when they are applied in long-term treatment for silicosis patients.

Our in vivo experiments indicated that, gefitinib and fostamatinib potently inhibited the level of p-EGFR and p-SYK respectively (Supplementary Fig. 6a, b), and effectively alleviated silica-induced diffuse alveoelitis revealed by histological examination (Supplementary Fig. 7a). Meanwhile, qPCR analysis showed that the levels of pro-inflammatory cytokines (such as *Interleukin-1β (Il-1β), Interleukin-6* (*Il-6*), and *Tumor necrosis factor α* (*Tnf-α*)) were markedly decreased in lung tissues (Fig. 5d) following drug treatment. Moreover, Van Gieson and sirius red staining data showed that collagen deposition of lung tissue was effectively suppressed by gefitinib or fostamatinib treatment in silicosis mice (Fig. 5f–g). Consistent with the pathological results, the mRNA expression of fibrotic genes, *fibronectin* 1 (*Fn-1*), and *collagen* I (*Col-1*) were also decreased in the inhibitor-treated mice (Fig. 5e). Taken together, these data indicated that gefitinib and fostamatinib treatment could suppress silicosis progression in mice.

### p-EGFR promoted fibronectin expression in fibroblasts and p-SYK enhanced pro-imflammatory cytokines production in macrophages

**Fig. 6:**
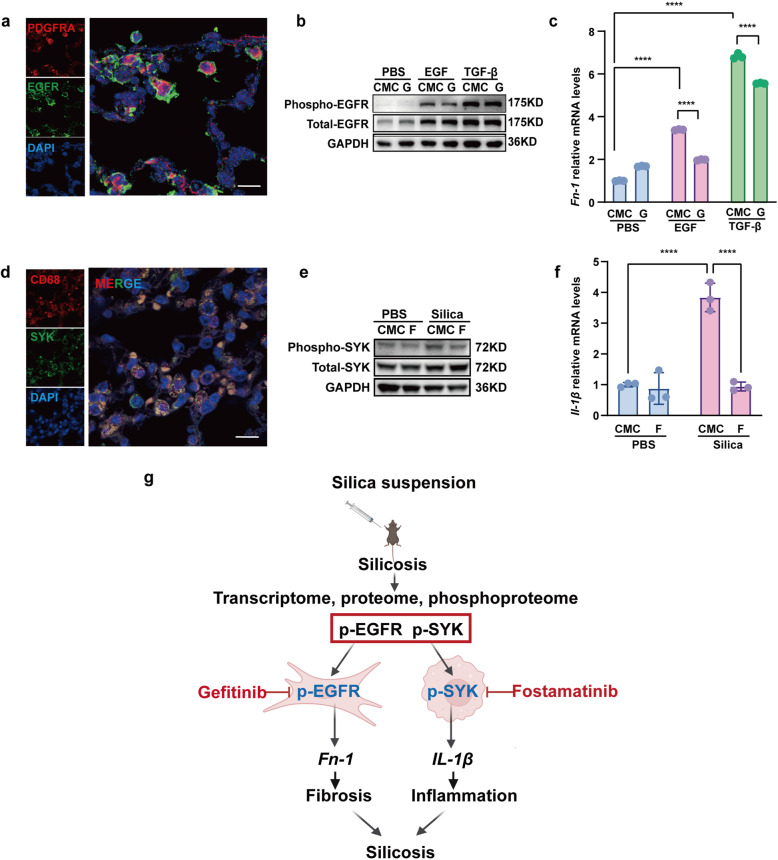
Gefitinib inhibited the profibrotic activity of lung fibroblasts while fostamatinib inhibited the pro-inflammatory activity of lung macrophages in silicosis. **a** EGFR and fibroblast marker expression in mouse lung tissue, fibroblasts were immunolabeled with anti-EGFR (green), anti-PDGFRA (red), and nuclei were immunolabeled with DAPI (blue). **b** Western blot analysis of EGFR expression and phosphorylation with control or EGF or TGF-β-treated ibroblasts cell line Mlg2908 lysates. **c** The relative mRNA levels of *Fn-1* in control and treated fibroblasts. **d** SYK and macrophage marker expression in mouse lung tissue, macrophages were immunolabeled with anti-CD68 (red), nuclei were immunolabeled with DAPI (blue) and SYK was immunolabeled with anti-SYK (green). **e** Western blot analysis of SYK expression and phosphorylation with treated and control cell lysates. **f** The relative mRNA levels of *Il-1β* in control and treated macrophages. **g** Graphical abstract of treatment process in cell experiments. All quantitative results were presented as mean ± SD. All experiment groups in panels c and f were compared by a two-way ANOVA followed by Bonferroni’s multiple comparisons test, *n* = 3 each group. ns: no significance; *p < 0.05, **p < 0.01, ***p < 0.001, ****p < 0.0001. PBS phosphate-buffered saline, G gefitinib, F fostamatinib

We further clarified the mechanisms of p-EGFR and p-SYK in the development of silicosis. We first examined the expression of EGFR in different cell types in mouse lung tissues. As shown in Fig. 6a, EGFR was mainly co-localized with the fibroblast marker PDGFRA. EGFR phosphorylation (p-EGFR) was reported to promote pulmonary fibrosis by activating downstream signaling pathways in fibroblasts. So we hypothesized that fibroblast-specific p-EGFR mediated lung fibrosis. To test this hypothesis, we treated the mouse lung fibroblast cell line Mlg2908 with EGF or TGF-β to simulate the pathological environment in vivo. The results showed that stimulation by EGF or TGFβ dramatically increased the expression of p-EGFR (Fig. 6b) and production of the extracellular matrix protein *Fn-1* (Fig. 6c). Blocking p-EGFR by gefitinib significantly inhibited *Fn-1* mRNA expression (Fig. 6c). These results suggested that phosphorylated EGFR regulated extracellular matrix protein production in fibroblasts, which was involved in silica-induced lung fibrosis.

Similarly, we first localized SYK protein expression in mouse lung tissues, and found that SYK co-localized with the macrophage marker CD68 (Fig. 6d). SYK has been reported to mediate macrophage functions in acute pulmonary inflammation^41^ and inflammation in pneumoconiosis.^42^ Meanwhile, alveolar macrophages (AMs) are the first immune cells to encounter silica nanoparticles in silicosis development. In this case, we explored the role of p-SYK in AMs during the progression of silicosis. In vitro, the murine AM cells (MH-S) were exposed to silica nanoparticles. As shown in Fig. 6e, f, silica exposure significantly elevated the expression of p-SYK and production of the pro-inflammatory cytokine *Il-1β*. Inhibiting p-SYK by fostamatinib dramatically downregulated *Il-1β* mRNA expression in MH-S cells (Fig. 6f). These in vitro data suggested phosphorylation of SYK mediated silica-stimulated pro-inflammatory cytokine production, which was involved in pulmonary inflammation.

## Discussion

Our current study presented a time-resolved multi-omics atlas of silicosis. Using this comprehensive dataset, phosphorylated EGFR and SYK were found to play significant roles in silicosis progression by regulating the extracellular matrix and production of inflammatory cytokines. Inhibition of p-EGFR and p-SYK by gefitinib and fostamatinib were found to effectively ameliorated the progression of silicosis via inhibiting p-EGFR mediated fibronectin production in fibroblasts and p-SYK mediated pro-inflammatory cytokines synthesis in macrophages.

The combination of multi-omics methods has been applied for potential drug target screening in cardiovascular,^37^ pulmonary,^38^ and neurodegenerative diseases.^39^ Despite intensive research, few efficient medications have been developed for silicosis. Thus, an unmet medical need for developing effective therapies in this patient population remains. As yet, most mechanistic studies of silicosis have been restricted to a few genes and specific pathways, leaving the etiopathology of silicosis poorly defined. Thus, no curative treatment has been approved for silicosis, and the currently used drugs cannot reverse the disease process.^1,43,44^ Recently, using transcriptome and metabolome datasets, our previous studies revealed several potential targets, such as FGF10, MUC5AC, PGD_2_ and TXA_2_. We also demonstrated ramatroban attenuated silicosis by inhibiting the receptors of PGD_2_ and TXA_2_.^19,45^ Of note, changes of these targets in our multi-omic dataset were in corroboration with our published data (Supplementary Fig. 5a, b). Furthermore, although limited, there is emerging in vitro evidence that expression of MUC5AC in COPD^46^ and COX2/PGE2 pathway in asthma^47^ are both regulated by p-EGFR, initiated by ligand-dependent activation. Based on these considerations, it is reasonable to speculate that p-EGFR may be a pleiotropic target and deserves further investigation. Notably, gefitinib and fostamatinib, inhibitors of p-EGFR and p-SYK, effectively attenuated silicosis, suggesting a promising strategy for silicosis. Consistent with our findings, EGFR is expressed and phosphorylated in fibroblasts, alveolar and airway epithelial cells as a key mediator of fibrogenesis^48–50^ and its inhibitors have been widely used in pulmonary fibrosis,^33^ renal fibrosis^51^ and cardiac remodeling.^52^ Similarly, there are accumulating data that SYK is implicated in the pathogenesis of fibrosis and related kinase inhibitors also show therapeutic potence.^21,37,53^

Both silicosis and IPF are common subtypes of progressive fibrosing interstitial lung diseases (PF-ILDs), usually exhibiting a severe loss of lung function, poor quality of life and early mortality.^3,18^ Evidently, silicosis and IPF show striking similarities in remodeling pattern of pulmonary structure in the early stage. Upon repetitive injury, the alveolar epithelial basal membrane loses integrity, and interstitial (myo)fibroblasts migrate across the fenestrations in these membranes into the intra-alveolar masses to produce excessive ECM.^18,54^ These intra-alveolar fibrotic lesions were then lined with epithelial cells and incorporated into alveolar walls, resulting in interstitial fibrosis.^10,54^ In terms of cell types involved in exacerbating fibrosis, while analogous, silicosis and IPF have some notable differences. For example, macrophages contributed to fibrosis in both silicosis and IPF. It is worth noticing that macrophages mediate interactions between epithelial cells and fibroblasts in IPF, while these cells are known to engulf silica particles and initiate the subsequent pro-inflammatory cascades in silicosis.^1,18,55^ Nevertheless, the common features of these two diseases still provide a strong basis for common therapeutic options. For example, nintedanib, a TKI inhibitor with FDA approval for IPF, has also shown positive results in silicosis in vivo.^56^ Consistently, our data found two novel TKI inhibitors for silicosis treatment, of which gefitinib was also previously used in IPF.^33^ Specifically, gefitinib strongly inactivated myofibrobalsts that acted as executors to elicit fibrosis via downregulating p-EGFR, while fostamatinib reduced lung inflammation by inhibiting p-SYK in macrophages. In summary, similarities of pathogenesis in fibrotic lung disorders indicated high potential of gefitinib and fostamatinib for silicosis treatment, but future studies are required.

In line with our findings, Ishii and colleagues reported that gefitinib ameliorated bleomycin-induced pulmonary fibrosis in mice at a dose of 200 mg/kg, even the dosage they chose was far higher than its clinical dosage in cancer.^33^ Also, Kudoh et.al. reconfirmed this case in radiation-induced lung fibrosis.^32^ In sharp contrast, several studies reached totally opposite conclusions that gefitinib augmented bleomycin-induced pulmonary fibrosis with possibilities that gefitinib exacerbated fibrosis via inhibiting the proliferation of epithelial cells or downregulating the expression of HSP70.^39,57^ Though not fully elucidated, reasons for this discrepancy might be partially due to the different mice strains. More importantly, clinical studies suggested that gefitinib was associated with acute lung injury and subsequent fibrosis in patients with NSCLC.^58^ However, the researchers also pointed out that the cases of fatal fibrosis in NSCLC patients caused by gefitinib were closely linked to previous alveolar damage from radiotherapy, pre-existed pulmonary comorbidities and infectious diseases.^58^ Additionally, considering that gefitinib conferred better clinical benefits in patients with mutated on exon 18, 19, and 21of EGFR,^59,60^ and the incidence of gefitinib-associated lung injury is higher in Japanese (5%) than that non-Janpanese population (0.8% globally).^50^ Thereby, it is reasonable to speculate such discrepancy may in part be attributed to differences in ethnicity and gene susceptibility. Also, clinical trials documented that both gefitinib and fostamatinib might also induce other side-effects, including diarrhea, drug-related hepatic and renal failure,^61^ as well as transaminitis and hyperbilirubinemia. These unfavorable effects should be weighed and balanced with the benefits when considering gefitinib and fostamatinib for mono- or combined therapies in silicosis. Together, although our results showed that gefitinib and fostamatinib had effective and safe profiles in silica-induced pulmonary fibrosis, a cautious interpretation is in future translational studies.

In this study, we mainly investigated drug targets in the consistently upregulated pathways, while other potential targets in the downregulated pathways remained to be explored in future studies (Supplementary Fig. 4). Further research is required to investigate these features and exploit them as biomarkers for diagnosis or targets for treatment.

In conclusion, our study has established a systematic atlas with multi-omics data from different stages of silicosis, and identified key signaling pathways and novel drug targets by integrative analysis. We have conducted preclinical study to provide proof-of-concept for the efficacy of two approved drugs, which may be re-purposed to treat silicosis. Combining multi-omics analysis with series of experiments, we have developed a pipeline for mechanism-based target identification and drug development for the treatment of silicosis.

## Materials and methods

### Animal model construction and drug treatment

All male C57BL/6J mice aged 10 weeks old were obtained from the Vital River Laboratory Animal Technology Co. Ltd. (Beijing, China) and all mice were housed under specific pathogen-free condition. The time-resolved silicosis mouse model was established as previously described.^22^ In previous studies, mice were exposed to silica at different doses for different lengths of time to simulate different stages of silicosis progression.^22,62,63^ In general, lower doses (4 mg/kg or more) were used to recapitulate the early inflammatory stages, while higher doses (up to 12 mg/kg) were used to construct models of the progressive or fibrotic stages. As silicosis patients are frequently diagnosed at their later stages, we used the high dose 12 mg/kg to simulate silicosis progression in mice. In brief, mice in silicosis group were received a single intratracheal instillation of 12 mg of silica in 40 μL of sterile PBS under pentobarbital anesthesia. The identical volume of PBS were administered to their control counterparts. To largely recapitulate the progress of silicosis, mice were sacrificed in different batches at 5 indicated time points, namely 0 week, 2 weeks, 4 weeks, 6 weeks and 10 weeks after silica exposure. The left and right lung lobes were collected to perform histological examination and multi-omics sequencing respectively.

For therapeutic experiments, control and silicosis mice were randomized into vehicle and treatment groups with either gefitinib (Macklin, G828597-5g, China) at a dose of 200 mg/kg or fostamatinib (ChemeGen, 901119-35-5, China) at a dose of 80 mg/kg. Based on our time-resolved atlas, we found persistent inflammation after 2 weeks (marked by IL-1/5/40 signaling pathways and IFN-γ/TNF-α responses), and progressive fibrosis after 4 weeks (marked by NF-kB activation pathways). Treatment thus begun at two weeks after silica exposure. Both gefitinib and fostamatinib were dissolved in 0.5% CMC solvent and were administered daily via oral gavage for two weeks. Ethics approval for all animal experiments was obtained from the Animal Care and Use Committee of Peking Union Medical College.

### Protein extraction

The sample was taken out from −80 °C, and an appropriate amount of tissue sample was weighed and placed into a mortar precooled with liquid nitrogen, and then fully grounded to powder by adding liquid nitrogen. The samples of each group were added with 4 times volume of lysis buffer (8 M urea, 1% protease inhibitor, 1% phosphatase inhibitor) for ultrasonic lysis. The remaining debris was removed by centrifugation at 12,000 × *g* at 4 °C for 10 min. Finally, the supernatant was collected and the protein concentration was determined with BCA kit.

### Trypsin digestion

For digestion, the protein solution was reduced with 5 mM dithiothreitol for 30 min at 56 °C and alkylated with 11 mM iodoacetamide for 15 min at room temperature in darkness. Then, the urea concentration of the sample was diluted to less than 2 M. After that, trypsin was added at 1:50 trypsin-to-protein mass ratio for the first digestion overnight and 1:100 trypsin-to-protein mass ratio for a second 4 h-digestion. Finally, the peptides were desalted and pending for subsequent analysis.

### Peptide fractionation

The peptide samples were then fractionated by high pH reverse-phase HPLC using Agilent 300 Extend C18 column (5 μm particles, 4.6 mm ID, 250 mm length). Briefly, peptides were first separated with a gradient of 8% to 27% acetonitrile in 10 mM ammonium bicarbonate pH 11 over 46 min into 36 fractions. Then, the peptides were combined into 12 fractions and dried by vacuum centrifuging.

### Phosphopeptide enrichment

Peptide mixtures were first incubated with Fe-IMAC microspheres suspension with vibration in loading buffer (50% acetonitrile/6% trifluoroacetic acid). The Fe-IMAC microspheres with enriched phosphopeptides were collected by centrifugation. To remove nonspecifically adsorbed peptides, the Fe-IMAC microspheres were washed with 50% acetonitrile/6% trifluoroacetic acid and 30% acetonitrile/0.1% trifluoroacetic acid sequentially. To elute the enriched phosphopeptides from the Fe-IMAC microspheres, elution buffer containing 10% NH_4_OH was added and the enriched phosphopeptides were eluted with vibration. The supernatant containing phosphopeptides was collected and lyophilized for LC-MS/MS analysis.

### LC-MS/MS analysis

The peptides were subjected to NSI source followed by tandem mass spectrometry (MS/MS) in Q Exactive HF-X (Thermo) coupled with EASY-nLC 1200 UPLC system. For phosphoproteome analysis, the gradient was comprised of an increasing gradient from 1% to 18% solvent B (0.1% formic acid in 90% acetonitrile) over 62 min, 18% to 32% in 20 min and climbing to 80% in 4 min then holding at 80% for the last 4 min. For proteome analysis, the gradient was reduced to 60 min. The electrospray voltage was applied at 2.2 kV. The intact peptides were detected in the orbitrap at a resolution of 60,000. Peptides were then selected for MS/MS with NCE 28 and the fragments were detected in the orbitrap at a resolution of 30,000. A data-dependent procedure that alternated between one MS scan followed by 20 MS/MS scans with 15 s dynamic exclusion. For phosphoproteome analysis, the automatic gain control (AGC) was set at 1E5, with an intensity threshold of 2E4 and a maximum injection time of 100 ms. For proteome analysis, the intensity threshold was set as 2.3E5 and a maximum injection time was 22 ms.

### Database search and data management

The resulting MS/MS data were processed using MaxQuant search engine (v.1.5.2.8). Tandem mass spectra were searched against Mus musculus (Swiss-Prot 201911 released) database concatenated with reverse decoy database. Trypsin/P was specified as cleavage enzyme allowing up to 2 missing cleavages. The mass tolerance for precursor ions was set as 20 ppm in the first search and 5 ppm in the main search, and the mass tolerance for fragment ions was set as 0.02 Da. Carbamidomethyl on Cys was specified as fixed modification, oxidation (M), acetylation (protein N-term), deamidation (NQ) and phosphorylation (STY) were specified as variable modifications. FDR was adjusted to <1% and minimum score for modified peptides was set to >40. Minimum peptide length was set at 7. For quantification method, match between run was enabled. All other parameters in MaxQuant were set to default values. Data management was performed using the Perseus software. One-way ANOVA test with Tukey post-hoc test adopted (adjusted *p* value < 0.05) was performed to identify differentially expressed proteins or phosphosites.

### RNA sequencing and data processing

The integrity and purity of RNA were checked prior to library preparations. A NEBNext^®^ UltraTM RNA Library Prep Kit for Illumina was used according to the manufacturer’s recommendations to create the sequencing libraries, which were then sequenced in 150-bp paired-end reads using an Illumina Hiseq platform (Novogene Co., Ltd, Beijing, China). The mouse reference genome and annotation files were downloaded from ftp://ftp.ensembl.org/pub/release-97. HISAT2 (v2.0.5) software^64^ was used to map the RNA-seq reads to the reference genome. After we got the read counts of genes using GenomicFeatures (v1.30.3) and GenomicAlignments (v1.14.2) packages,^65^ we performed differential analysis between any two groups using DESeq2 (v1.18.1) package.^66^ The cutoff of adjusted *p-value* < 0.05 with Benjamini & Hochberg correction was used to select differential genes, and all the differential genes were merged to perform downstream pattern analysis.

### Bioinformatic analysis

Differentially expressed features were identified from each omic dataset as referred in database searching and data management, as well as RNA sequencing and data processing. Principal component analysis (PCA) was performed with all identified features to explore the largest sources of variation within each omics dataset. Unsupervised hierarchical clustering were performed within pheatmap (v. 1.0.12) through R package (v. 3.6). The discrete clusters of features with similar expression changes for omics data were identified on fuzzy c-means algorithm implemented with Mfuzz (v. 2.48.0) through R package (v. 3.6). The pathway enrichment of clustered features was achieved with metacore (Clarivate Accelerating innovation) based on canonical signaling pathways database from Clarivate Analytics. Weighted *p* value of overlapped pathways (enriched with features from consistently upregulated or downregulated clusters) was calculated as the products of *p-value* enriched in three omics datasets. metascape (v. 3.5) was applied for integrated analysis of multi-omics datasets. Consistently changed features were conversed as Entrez ID in metascape then the correlation of features or their annotation from different clusters was visualized with Circos (v. 0.69-9). Enrichment clustering was applied in metascape based on integrated ontology sources including GO, Kyoto Encyclopedia of Genes and Genomes (KEGG), and MSigDB,^67^ and related data were visualized as network with Cytoscape (v. 3.8.1).

### Western blot

Mouse lung tissue samples were lysed in RIPA buffer (50 mM Tris–HCl (pH 7.4), 150 mM NaCl, 1% Triton X-100, 1 mM EDTA, 10% Glycerol), and protease/phosphatase inhibitors, 0.3 mM dithiothreitol, 5 mM nicotinamide and 1 mM sodium butyrate were added to the buffer. Western blots were performed according to standard protocols. Antibodies and the dilutions were anti-GAPDH (10494-1-AP, Proteintech, 1:10000), anti-β-actin (sc-5286, Santa Cruz, 1:10000), anti-Syk (13198, Cell Signaling Technology, 1:1000), anti-Phospho-Syk (14140, Cell Signaling Technology, 1:1000), anti-EGFR (PA1-1110, Invitrogen, 1:1000), anti-Phospho-EGFR (8808, Cell Signaling Technology, 1:1000).

### Immunohistochemistry

Paraffin tissue sections were incubated at 65 °C for 20 min then treated with dewax solution using concentration gradients of xylene and alcohol. After washing with PBS, tissue sections were treated with citrate buffer (pH 6.0), they were exposed to heat-induced epitope retrieval (100 °C) for 3 min and heating for 20 min. After treatment with 3% hydrogen peroxide for 10 min and goat serum at 37 °C for 30 min, the tissue sections were incubated with primary antibodies against anti-Collagen I (Abcam, ab254113, 1:200) at 4 °C overnight. The tissue sections were subsequently incubated with secondary antibodies (Goat anti-Rabbit, PV-6001) at room temperature for 60 min. After washing with PBS, these sections were stained using 3,3-diaminobenzidine (DAB, ZSGB-BIO, ZLI9017). The staining was terminated when the tissue sections were brown. Subsequently, all the tissue sections were counterstained with hematoxylin for 12 s. Finally, these sections were dehydrated with ethanol and toluene then sealed with neutral gum. Photographs were taken under microscope and the percentage of positive area was taken as statistical basis.

### Histological staining and analysis

Hematoxylin-eosin (HE), Van Gieson, masson and sirius red staining were performed in the lung tissues of mice. Van Gieson staining was carried out according to the instructions of the kit (BASO, BA4084A). Sirius red staining was performed by using sirius red staining kit (RS1240, G-CLONE, Beijing, China). The experimental steps was followed by the manufacturer’s protocol. The percentage of fibrotic area was measured by Image J pro with Van Gieson staining and sirius red staining data.

### Statistical analysis

Normality of quantitative data was first analyzed using Graphpad Prism 9.0.0 (GraphPad Software, Inc). Comparisons among groups in experiments data were performed by a two-way ANOVA followed by Bonferroni’ s multiple comparisons test, ns: no significance; *p < 0.05, **p < 0.01, ***p < 0.001, ****p < 0.0001.

## Supplementary information

Supplementary Materials

Supplementary table S1

Supplementary table S2

Supplementary table S3

Supplementary table S4

Supplementary table S5

Supplementary table S6

Supplementary table S7

Supplementary table S8

Supplementary table S9

Supplementary table S10

## Data Availability

The RNA-seq data of this study has been deposited in the Genome Sequence Archive (GSA) (https://bigd.big.ac.cn/) with the Accession number: CRA005925. The mass spectrometry proteomics data have been deposited to the ProteomeXchange Consortium (http://proteomecentral.proteomexchange.org) via the iProX partner repository^68^ with the dataset identifier PXD027693.
