# Supplementary material for: Gefitinib and fostamatinib target EGFR and SYK to attenuate silicosis: a multi-omics study with drug exploration
Source: Signal Transduct Target Ther. 2022 May 13;7:157. doi: 10.1038/s41392-022-00959-3 (PMC9098425; doi:10.1038/s41392-022-00959-3)
Supplement: Supplementary file 1 — Supplementary Materials [file 41392_2022_959_MOESM1_ESM.docx]

Supplementary Materials for

Gefitinib and Fostamatinib target EGFR and SYK to attenuate silicosis: a multi-omics study with drug exploration

Mingyao Wang^1,2*^, Zhe Zhang^3,4,5*^, Jiangfeng Liu^1*,+^, Meiyue Song^1,6*^, Tiantian Zhang^1*^, Yiling Chen^1,7^, Huiyuan Hu^1,7^, Peiran Yang^1^, Bolun Li^1^, Xiaomin Song^1^, Junling Pang^1^, Yanjiang Xing^1^, Zhujie Cao^1^, Wenjun Guo^1^, Hao Yang^8^, Juntao Yang^1+^, Jing Wang^1+^, Chen Wang^1^

Correspondence to: [ljf@pumc.edu.cn](mailto:ljf@pumc.edu.cn)，yangjt@pumc.edu.cn，wangjing@ibms.pumc.edu.cn

**This PDF file includes:**

Supplementary figure S1-S7

Supplementary table S1-S10 (See attached Excel files)

**Supplementary Figures**

**
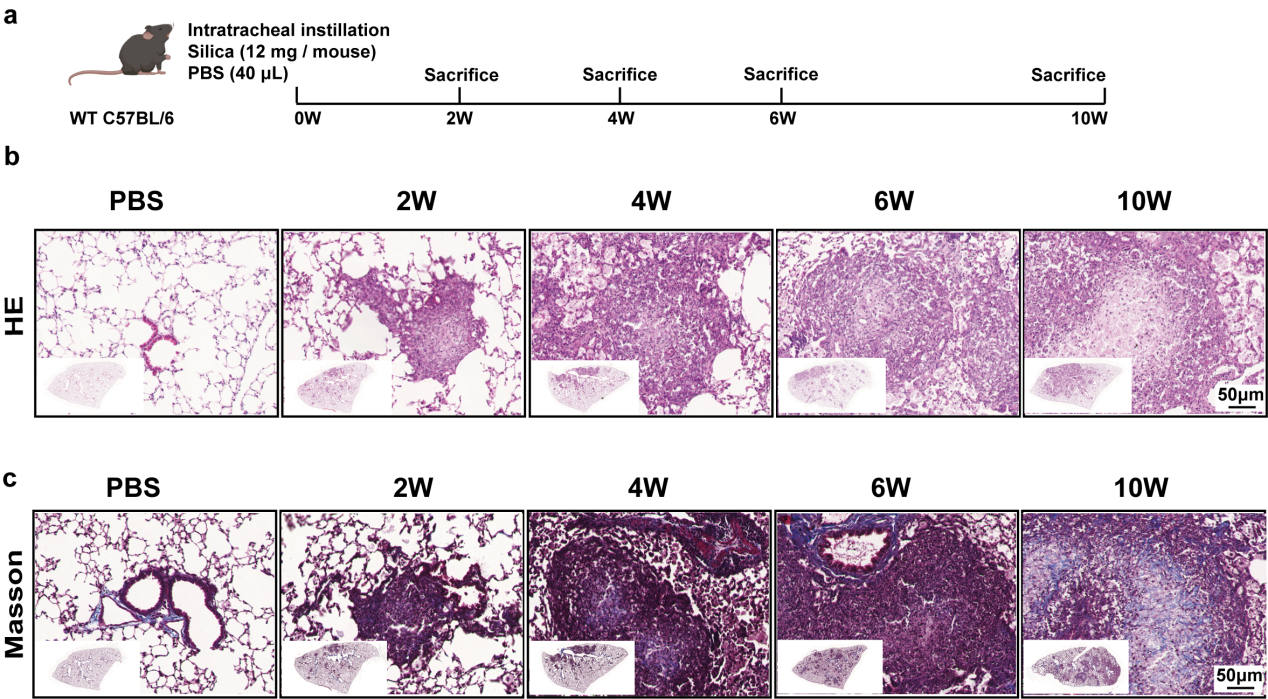
**

**Figure S1. Pathophysiological changes of silicosis mouse model in different stages.**

**(a)** Schematic diagram of time points to collect lung tissues for multi-omics sequencing. Representative images of **(b)** HE staining and **(c)** Masson staining in lung tissues from silicosis mouse model in different stages, n = 4 for each group. 2W, 2 weeks; 4W, 4 weeks; 6W, 6 weeks, 10W, 10 weeks.

**
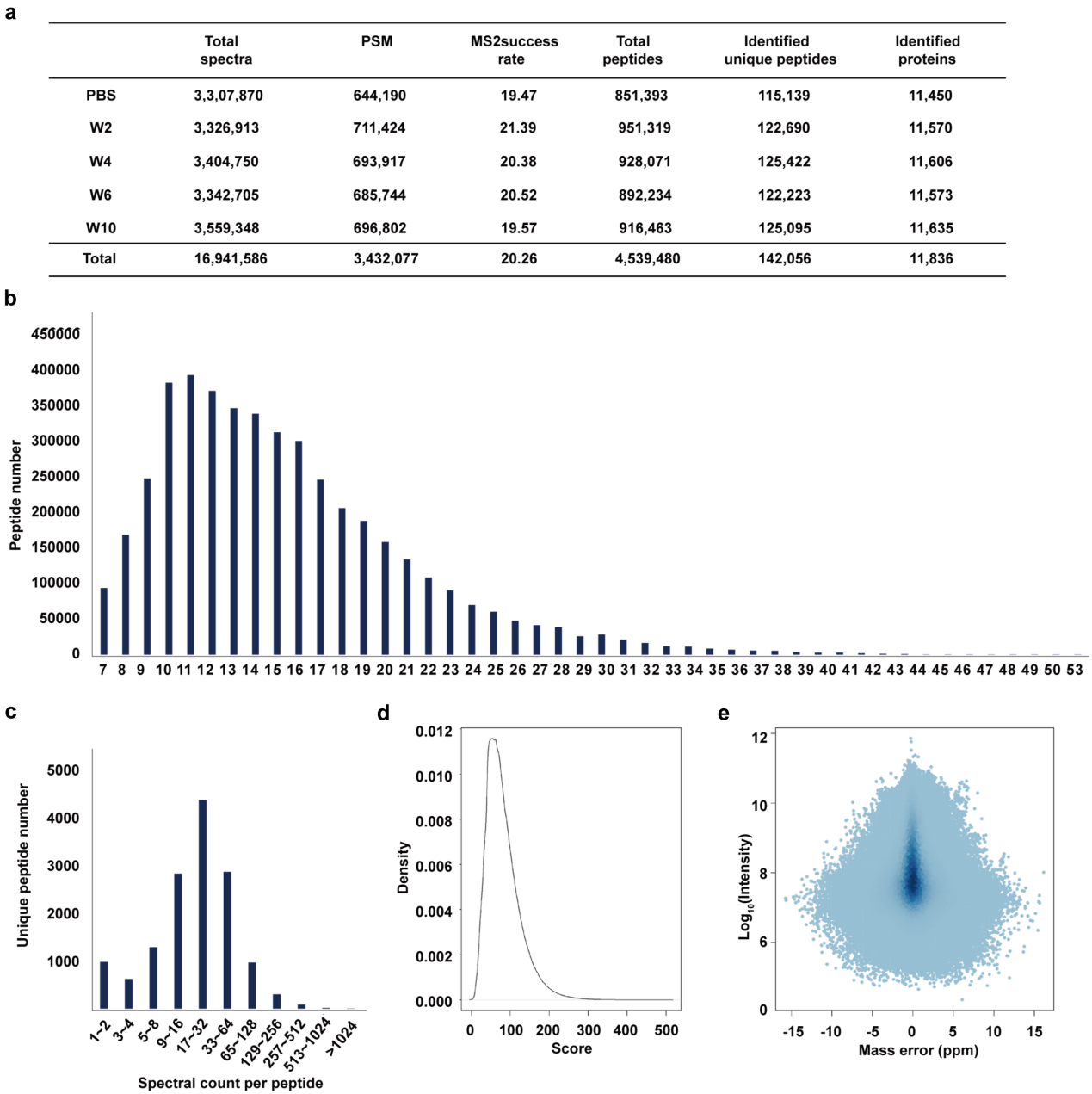
Figure S2.** **Quality control of LC-MS/MS in label-free quantitative proteome.**

**(a)** Identified spectra and peptide for label-free quantitative proteins. **(b)** Peptide length distribution for all label-free quantitative proteins. **(c)** Histogram of peptide-to-spectrum matches (PSMs) per unique identified peptide. **(d)** Andromeda score distribution for all identified peptides. **(e)** Mass error distribution of all identified peptides.

**
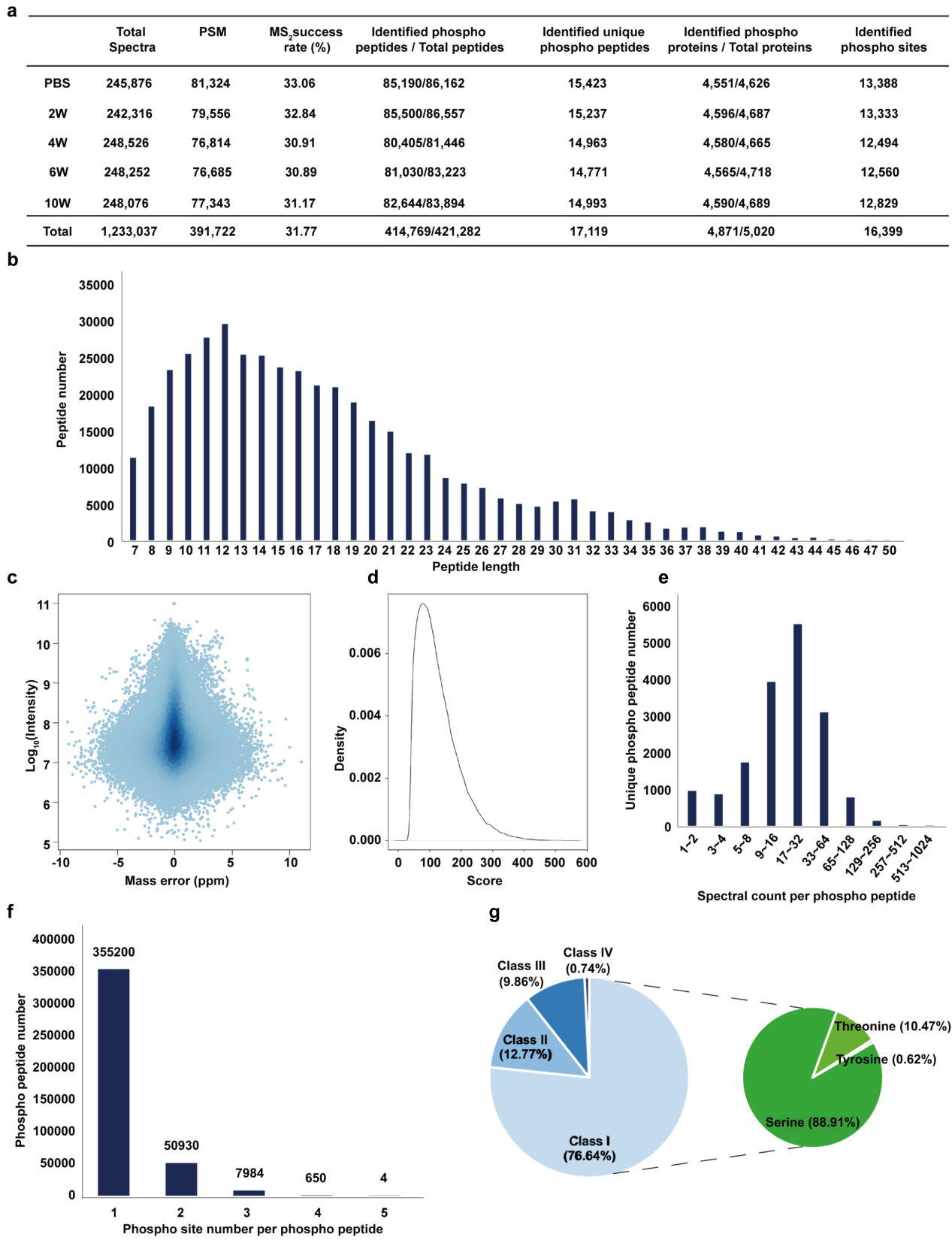
Figure S3.** **Quality control of LC-MS/MS in label-free quantitative phosphoproteome.**

1. Identified spectra and peptide for label-free quantitative proteins. **(b)** Peptide length distribution for all label-free quantitative proteins. **(c)** Mass error distribution of all phosphorylated peptides. **(d)** Andromeda score distribution for all identified peptides. **(e)** Histogram of peptide-to-spectrum matches (PSMs) per unique identified peptide. **(f)** Distribution of unique phosphorylation peptides number on the spectral count of each phosphorylated peptides. **(g)** Venn diagram of modification type distribution of phosphorylation sites.

**
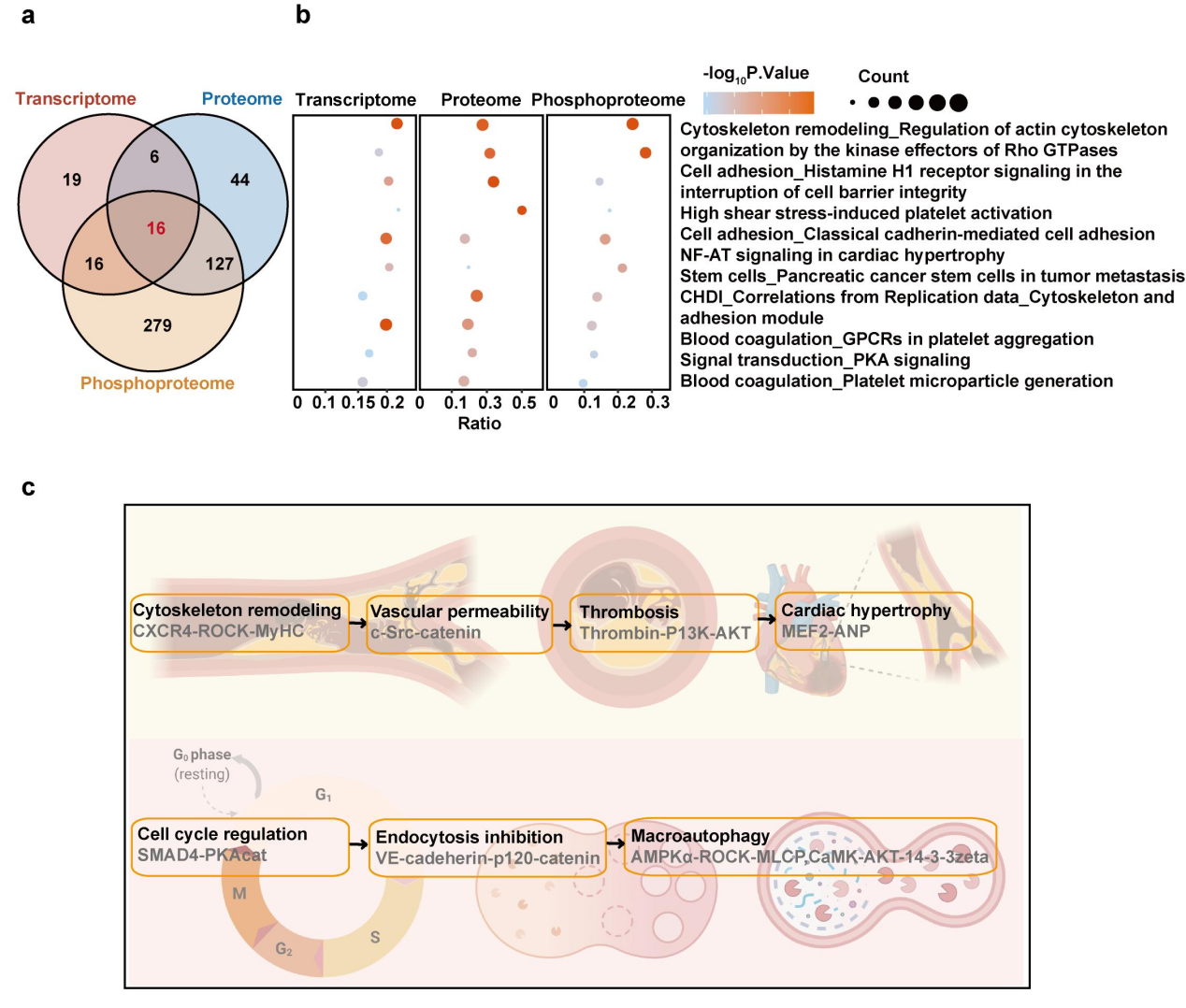
**

**Figure S4. Combined pathway analysis with metacore for down-regulated features.**

1. Shared pathways of down-regulated modules above obtained from metacore enrichment with the cut-off adjusted *p-value* < 0.05. **(b)** Bubble plot of top10 shared pathways in **(a)**, the color represents adjusted *p-value* and bubble size represents counts of genes in the top10 enriched pathways. **(c)** Illustration of the proposed underlying mechanisms involved in silicosis progression based on evidence from the multi-omics enrichment analysis.


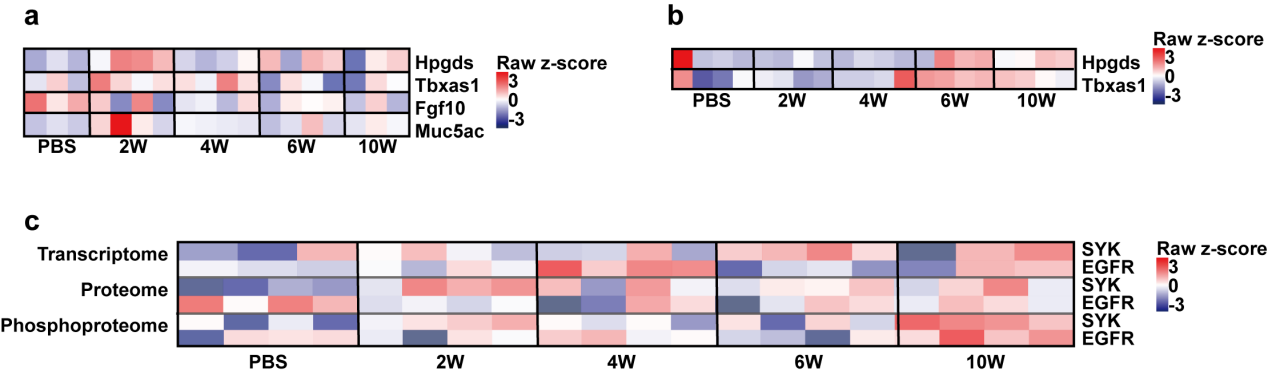


**Fig. S5. Heatmaps of** **(a)** *Hpgds*, *Tbxas1*, *Fgf10* and *Muc5ac* in mRNA level, **(b)** HPGDS and TBXA1 in protein level, **(c)** SYK and EGFR expression in mRNA, protein and phosphorylation levels. 2W, 2 weeks; 4W, 4 weeks; 6W, 6 weeks, 10W, 10 weeks.


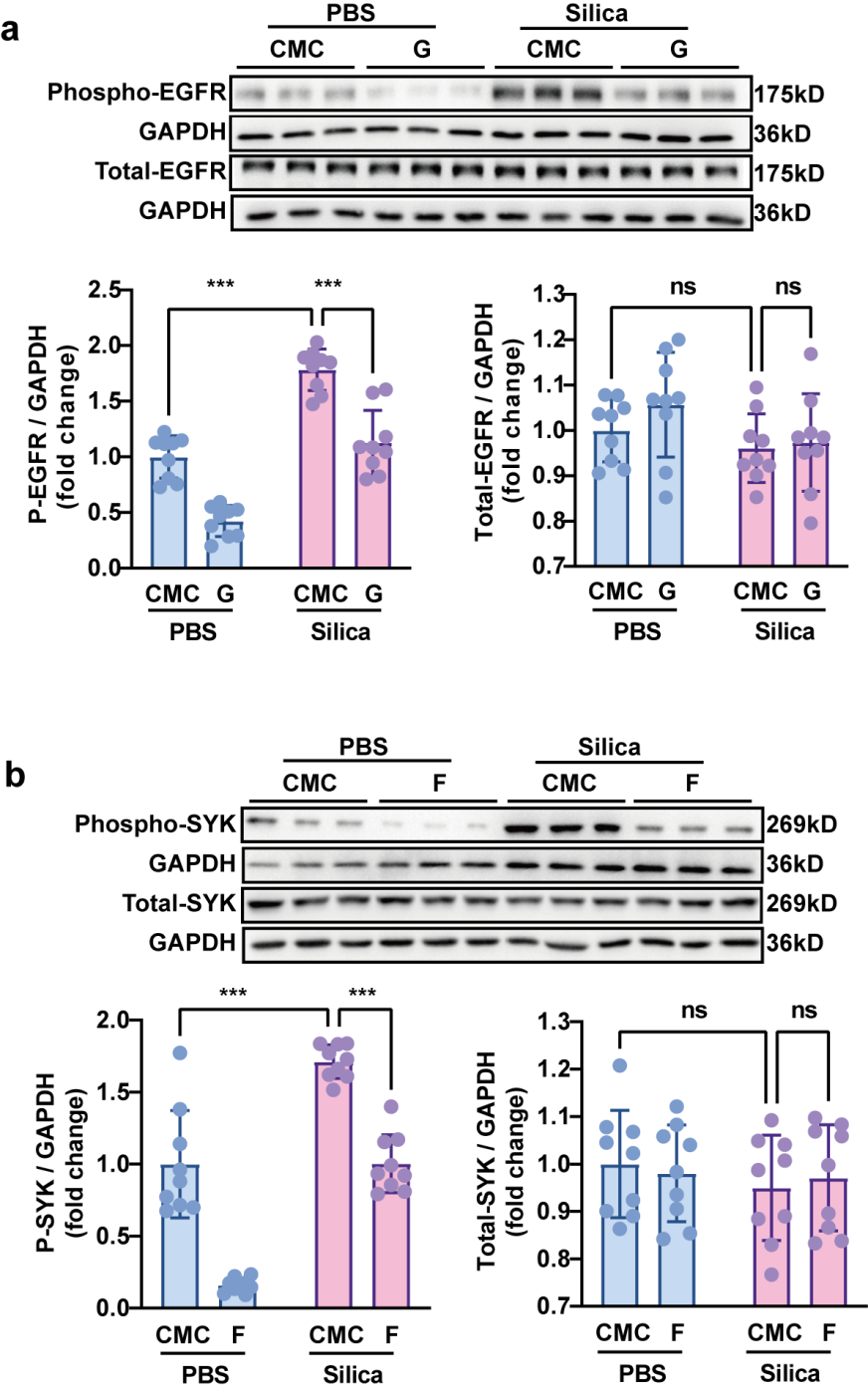


**Figure S6. Gefitinib and fostamatinib treatment suppressed p-EGFR and p-SYK expression in silicosis mice.**

**(a)** Western blot analysis indicating the effects of gefitinib treatment on phospho-EGFR and total-EGFR in silicosis and control mice. **(b)** Western blot analysis indicating the effects of fostamatinib treatment on phospho-SYK and total-SYK in silicosis and control mice. All the experimental groups in **(a)** and **(b)** were compared by unpaired t-test (the result of p-SYK was followed by Mann Whitney test), PBS group: n = 6 each group; Silica group: n = 6 each group; ns: no significance, **p* < 0.05, ** *p* < 0.01, ****p* < 0.001. PBS, phosphate-buffered saline; G, gefitinib; F, fostamatinib.


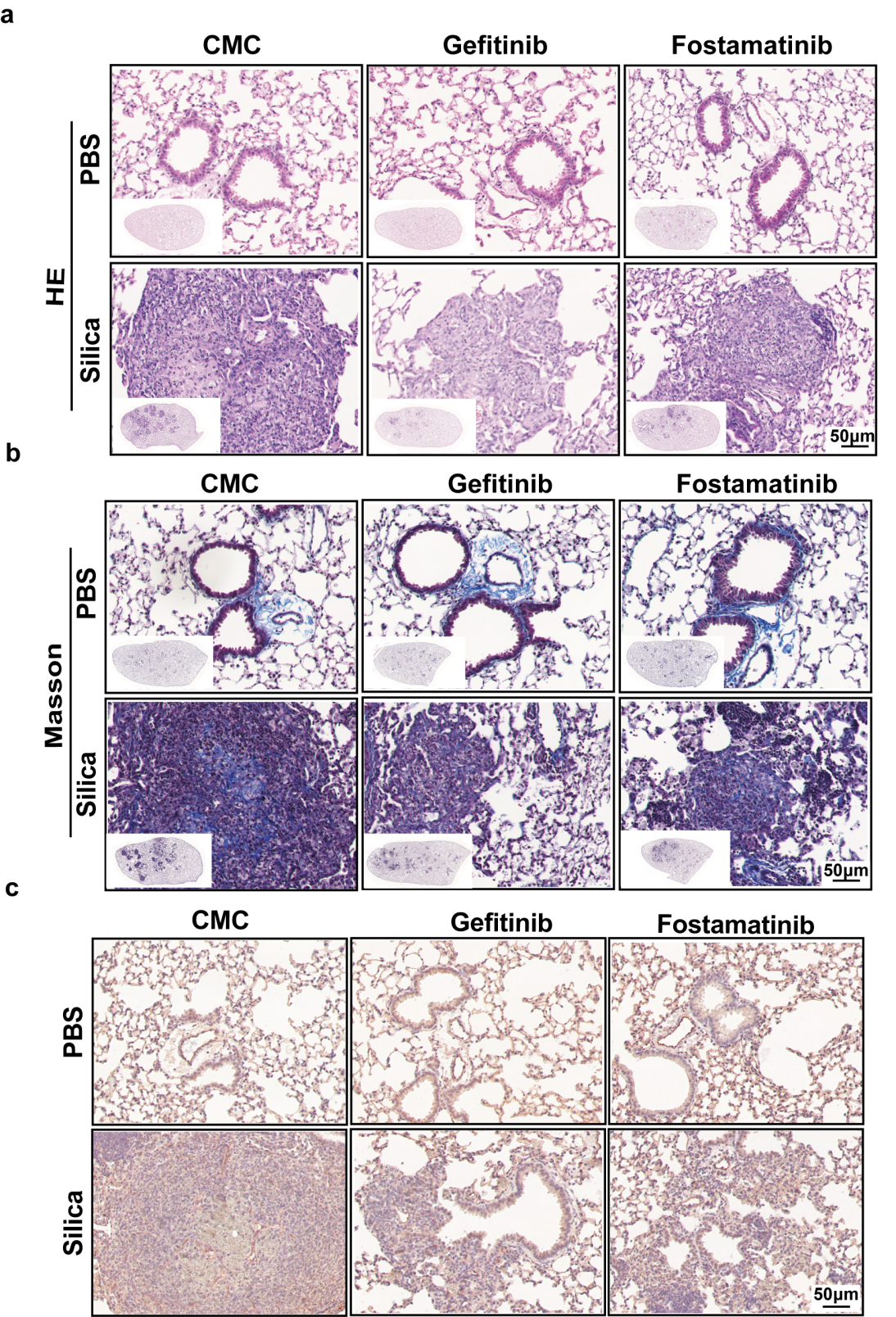


**Figure S7. Gefitinib and fostamatinib treatment suppressed pulmonary inflammation and fibrosis progression.**

Representative images of **(a)** HE, **(b)** Masson and **(c)** Collagen I immunohistochemical staining, and the scale bar indicated 50 μm. PBS group: n = 9 each group; Silica group: n = 9 each group. PBS, phosphate-buffered saline; CMC, carboxymethyl cellulose; G, gefitinib; F, fostamatinib.
